# Supplementary material for: Can network science reveal structure in a complex healthcare system? A network analysis using data from emergency surgical services
Source: BMJ Open. 2020 Feb 9;10(2):e034265. doi: 10.1136/bmjopen-2019-034265 (PMC7044848; doi:10.1136/bmjopen-2019-034265)
Supplement: Supplementary data [file bmjopen-2019-034265supp001.pdf]

Appendix – Supplementary table

Abbreviation Ward area	Ward area
AE or A&E	Emergency department
Angio	Angiography
CDU	Clinical decision unit (short stay)
Clinic	Referrals and appointments within the hospital
CT	Computer tomography
Echo	Trans-thoracic echocardiography
HDU	High-dependency unit
ICU	Intensive care
IR	Interventional radiology
MRI	Magnetic resonance
NCCU	Neuro-critical care
neuro theatre	Neuro-surgical theatres
NP	Neuro-physiology
PET	Positron emission tomography
PICU	Paediatric intensive care
Rehab	Rehabilitation ward
TOE	Trans-oesophageal echocardiography
US	Ultrasound
VA	Vascular access services
XR	Radiology (X-ray department)

Abbreviations used throughout the document for ward areas and services.
